# Supplementary material for: Splice-Junction-Based Mapping of Alternative Isoforms in the Human Proteome
Source: Cell Rep. Author manuscript; Available in PMC 2020 Jan 15. (PMC6961840; doi:10.1016/j.celrep.2019.11.026)
Supplement: 2 [file NIHMS1546469-supplement-2.zip › DF1/PXD009737/TestisGluC/TestisGluC_8_EPB41L3_TTSTTTTTHITKALAQAIKE.pdf]

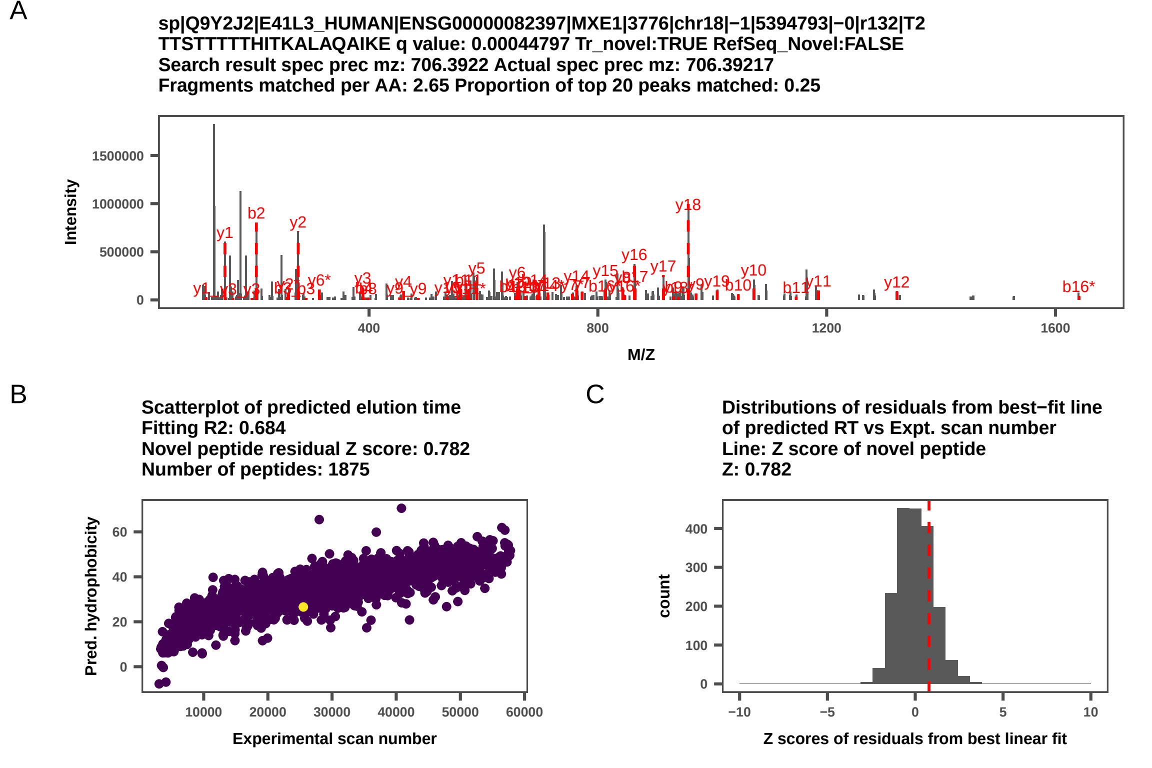
A
sp|Q9Y2J2|E41L3_HUMAN|ENSG00000082397|MXE1|3776|chr18|−1|5394793|−0|r132|T2
TTSTTTTTHITKALAQAIKE q value: 0.00044797 Tr_novel:TRUE RefSeq_Novel:FALSE
Search result spec prec mz: 706.3922 Actual spec prec mz: 706.39217
Fragments matched per AA: 2.65 Proportion of top 20 peaks matched: 0.25
Intensity
1500000
1000000
500000
0
y1
b2
y2
y18
y16
y17
y5
y6*
y10
y11
y12
b16*
b11
b10
400
800
1200
1600
M/Z
B
Scatterplot of predicted elution time
Fitting R2: 0.684
Novel peptide residual Z score: 0.782
Number of peptides: 1875
Pred. hydrophobicity
60
40
20
0
10000
20000
30000
40000
50000
60000
Experimental scan number
C
Distributions of residuals from best−fit line
of predicted RT vs Expt. scan number
Line: Z score of novel peptide
Z: 0.782
count
400
300
200
100
0
−10
−5
0
5
10
Z scores of residuals from best linear fit
